# Assessing seasonal and weather effects on depression and physical activity using mobile health data

**DOI:** 10.1038/s44184-025-00125-x

**Published:** 2025-04-18

**Authors:** Yuezhou Zhang, Amos A. Folarin, Yatharth Ranjan, Nicholas Cummins, Zulqarnain Rashid, Pauline Conde, Callum Stewart, Shaoxiong Sun, Srinivasan Vairavan, Faith Matcham, Carolin Oetzmann, Sara Siddi, Femke Lamers, Sara Simblett, Til Wykes, David C. Mohr, Josep Maria Haro, Brenda W. J. H. Penninx, Vaibhav A. Narayan, Matthew Hotopf, Richard J. B. Dobson, Abhishek Pratap, RADAR-CNS consortium, Yuezhou Zhang, Amos A. Folarin, Yatharth Ranjan, Nicholas Cummins, Zulqarnain Rashid, Pauline Conde, Callum Stewart, Shaoxiong Sun, Srinivasan Vairavan, Faith Matcham, Carolin Oetzmann, Sara Siddi, Femke Lamers, Sara Simblett, Til Wykes, David C. Mohr, Josep Maria Haro, Brenda W. J. H. Penninx, Vaibhav A. Narayan, Matthew Hotopf, Richard J. B. Dobson

**Affiliations:** 1 Department of Biostatistics & Health Informatics, Institute of Psychiatry, Psychology and Neuroscience, King’s College London, London, UK; 2 Institute of Health Informatics, University College London, London, UK; 3 NIHR Biomedical Research Centre at South London and Maudsley, NHS Foundation Trust, London, UK; 4 Health Data Research UK London, University College London, London, UK; 5 NIHR Biomedical Research Centre at University College London Hospitals, NHS Foundation Trust, London, UK; 6 Department of Computer Science, University of Sheffield, Sheffield, UK; 7 Janssen Research and Development LLC, Titusville, NJ USA; 8 Department of Psychological Medicine, Institute of Psychiatry, Psychology and Neuroscience, King’s College London, London, UK; 9 School of Psychology, University of Sussex, Falmer, UK; 10 Teaching Research and Innovation Unit, Parc Sanitari Sant Joan de Déu, Fundació Sant Joan de Déu, Barcelona, Spain; 11 Centro de Investigación Biomédica en Red de Salud Mental, Madrid, Spain; 12 Department of Psychiatry, Amsterdam UMC, Vrije Universiteit, Amsterdam, The Netherlands; 13 Mental Health Program, Amsterdam Public Health Research Institute, Amsterdam, The Netherlands; 14 Department of Psychology, Institute of Psychiatry, Psychology and Neuroscience, King’s College London, London, UK; 15 South London and Maudsley NHS Foundation Trust, London, UK; 16 Center for Behavioral Intervention Technologies, Department of Preventive Medicine, Northwestern University, Chicago, IL USA; 17 Department of Psychiatry, University of Oxford, Oxford, UK; 18 Boehringer Ingelheim Pharmaceuticals, Inc., Ridgefield, CT USA; 19 University of Washington, Seattle, WA USA

**Keywords:** Data mining, Statistical methods, Depression, Climate-change impacts

## Abstract

Seasonal and weather changes can significantly impact depression severity, yet findings remain inconsistent across populations. This study explored depression variations across the seasons and the interplays between weather changes, physical activity, and depression severity among 428 participants in a real-world longitudinal mobile health study. Clustering analysis identified four participant subgroups with distinct patterns of depression severity variations in 1 year. While one subgroup showed stable depression levels throughout the year, others peaked at various seasons. The subgroup with stable depression had older participants with lower baseline depression severity. Mediation analysis revealed temperature and day length significantly influenced depression severity, which in turn impacted physical activity levels indirectly. Notably, these indirect influences manifested differently or even oppositely across participants with varying responses to weather. These findings support the hypothesis of heterogeneity in individuals’ seasonal depression variations and responses to weather, underscoring the necessity for personalized approaches in depression management and treatment.

## Introduction

Depression, recognized as the most prevalent mental disorder worldwide, is a leading cause of disability^1^. Despite its substantial economic and social burden^2^, the underlying etiology, pathophysiology, and effective treatments remain partially understood^3–5^. Previous research has shown that the variability of environmental factors (e.g., seasons and weather conditions)^6–9^ and individual behaviors (e.g., physical activity, social interaction, and sleep)^10–14^ can impact depression symptom severity and its long-term trajectory. However, the impact of such external environmental factors on depression may manifest differentially across individuals^15–18^. Furthermore, there could be potential interplays between environmental factors, behaviors, and depression severity (e.g., weather may affect depression status, which in turn impacts physical activity) that are not fully understood so far. Further research is needed to understand how the short- and long-term changes in weather and real-world behaviors are interconnected with depression symptom severity and may impact individuals differently.

Previous studies have shown the significant impact of weather variations and seasonality on individual depression severity^19–21^. Seasonal Affective Disorder (SAD) is conceptualized as a subtype of depression characterized by different symptomatology and marked seasonal patterns of recurrent depressive episodes^19,20^. However, the literature shows notable inconsistencies in seasonal variation at the population level. While some studies report increased depression symptoms in winter^20,22,23^, others demonstrated peaks in depression severity during spring^21,24,25^, summer^21,26^, and autumn^24^, with still others failing to find any significant seasonal effects^27,28^. Regarding weather-related factors, some studies linked severe depressive symptoms to higher rainfall, lower temperature, reduced sunlight, and overcast conditions^21,29^. In contrast, some studies have found contradictory findings^30,31^ or no significant correlations^32–34^. The heterogeneity of individual affective responses to varying weather conditions within study cohorts might be one of the potential reasons for these inconsistent findings^33^. For example, if a subgroup of individuals responds positively to changes in weather or season (e.g., temperature) while another subgroup responds negatively or shows no response, the overall cohort level effect may be non-significant^33^. Furthermore, even SAD diagnoses are differentiated between winter and summer SAD^35,36^, indicating individual differences in responses to daylight exposure^33^. Therefore, it is essential to investigate different seasonal variation patterns of depression and different subtypes of responses to weather changes within populations.

Varying weather patterns can also significantly impact real-world behaviors, especially physical activity^37,38^. For example, research has shown a positive correlation between physical activity levels and both ambient temperature and day length, while a negative correlation exists with precipitation and wind speed^38–40^. In addition, physical activity and depression are negatively associated in a bidirectional manner. For example, depression may lead to decreased levels of physical activity, while reduced physical activity is also a known risk factor for depression^11,41–43^. Consequently, the impact of weather on depression severity and physical activity may be interlinked (see Supplementary Fig. 1 for a schematic diagram). For instance, weather conditions may directly affect physical activity levels or indirectly influence them by lowering mood. Such interplays between weather and physical activity on behavior can be systematically explored with real-world long-term observational data using mediation analysis. The analysis provides insights into how one variable can affect another through the intermediary role of a mediator^44^. A comprehensive understanding of how weather affects both depression and physical activity could refine personalized prevention strategies for depression, such as tailored use of exercise^43^.

Despite the importance of these interconnected relationships, to our knowledge, no studies have explored the mediating effects between depression, physical activity, and environmental factors such as weather. Furthermore, most previous research on weather and depression has been cross-sectional, focusing on broad cohort associations^6,21,29,31^, which limits understanding of individual differences and within-individual associations^32^. Additionally, many previous studies have relied on participants recalling their emotional and behavioral states over months or years, which may introduce the subjective recall bias^22,45,46^. Longitudinal observational studies that use mobile technologies offer a cost-efficient method to monitor participants’ behaviors, health status, and environmental variables over a longer term^47,48^.

To address the research gaps, this research leveraged data from the Remote Assessment of Disease and Relapse Major Depressive Disorder (RADAR-MDD) study, one of the first longitudinal observational studies to collect over a year’s mobile health data from a depression cohort in real-world settings^49^. The primary aim was to explore the mediating effects between weather conditions, physical activity, and depression severity, and to identify if there are subgroups of participants who exhibit distinct mediating effects. The secondary aim was to investigate potential patterns of depression variations across the seasons in the 1-year observation period in the dataset.

## Methods

### Participants and settings

This study leveraged data from the RADAR-MDD research program, which explored the effectiveness of remote mobile technologies for monitoring depression and predicting relapse in MDD^49^. The RADAR-MDD study recruited 623 participants from three study sites in the United Kingdom, Spain, and the Netherlands and followed them for up to 2 years^50^. Recruitment spanned November 2017 to June 2020, with data collection concluding in April 2021^50^. Due to rolling enrollment, the follow-up duration varied from 11 months to 24 months^50^. Utilizing the RADAR-base open-source platform, the RADAR-MDD program concurrently gathered both active (e.g., questionnaires) and passive (e.g., smartphone and Fitbit device) data^51^.

The RADAR-MDD protocol was co-developed with a patient advisory board (PAB), who shared their opinions on several aspects of the study, including the choice and frequency of survey measures, the usability of the study app, participant-facing documents, selection of optimal participation incentives, and the deployment of wearable device as well as the data analysis plan.

Ethical approvals were obtained from the Camberwell St. Giles Research Ethics Committee (17/LO/1154) in the UK, the Fundacio Sant Joan de Deu Clinical Research Ethics Committee (CI: PIC-128-17) in Spain, and the Medische Ethische Toetsingscommissie VUmc (2018.012–NL63557.029.17) in the Netherlands.

### Measures

#### Depression severity

The severity of participants’ depression symptoms was measured biweekly via smartphones using the 8-item Patient Health Questionnaire (PHQ-8)^52^. The PHQ-8 contains eight questions, with the total score ranging from 0 to 24, indicating increasing severity^52^.

#### Seasons

The seasons were divided based on the meteorological season calendar: spring begins on March 1, summer on June 1, autumn on September 1, and winter on December 1^31^. Each PHQ-8 questionnaire was assigned a season based on its completion date.

#### Weather variables

Throughout the RADAR-MDD study period, the city information relating to the participant’s location was recorded at the time of PHQ-8 assessment completion via the study app^51^. Utilizing coarse geospatial information, individual-level weather data were retrospectively sourced from the OpenWeather historical API (https://openweathermap.org/api/). We extracted the daily averages for several key meteorological parameters—ambient temperature (in Celsius), atmospheric pressure (in hPa), humidity (%), wind speed (in meters/sec), cloudiness (%), and day length (the time between sunrise and sunset, measured in hours)—specific to the dates on which the PHQ-8 questionnaires were completed.

#### Physical activity

As part of the RADAR-MDD study, participants were instructed to wear a Fitbit wristband to monitor their daily behaviors^49^. The total step count per day as measured by Fitbit was used as an approximate surrogate measure for the daily physical activity level. Since the PHQ-8 is designed to evaluate depression severity over the past 2 weeks^52^, we then calculated the average of these daily total step counts within a 2-week window prior to each PHQ-8 assessment. This approach linked the participant’s physical activity level with both the depression assessment and weather conditions.

#### Covariates

Besides weather and seasonal factors, participants’ depression severity and physical activity levels can be influenced by several other variables, such as socio-demographics^53,54^. To control the confounding effects, we considered age, gender, years of education, parental status, employment status, marital status, annual income level, and study site, collected at the time of enrollment in the study, as covariates in subsequent mediation analysis. Furthermore, since the data collection occurred during the COVID-19 pandemic, we also included a binary covariate representing the presence of a national lockdown to partially account for the potential effects of COVID-19 restrictions on participants’ physical activity and mental health^55,56^.

### Data inclusion criteria

To ensure data integrity, we filtered out incomplete submissions of the PHQ-8 questionnaire. Since this study included analyzing seasonal variations, participants were excluded from the study if they failed to complete the PHQ-8 questionnaire during any given season. Additionally, to align follow-up durations across participants, we only included data from the first year for those who had participated for more than 1 year.

### Clustering analysis—depression severity variations across the seasons in the 1-year observational period

We calculated the average PHQ-8 scores for each participant for each of the four seasons separately and then mean-centered these seasonal averages by subtracting the participant’s overall mean PHQ-8 score (the average of the whole year). Each participant was represented by a vector containing four elements corresponding to each of the four seasons. To identify distinct seasonal patterns within the cohort, we utilized the K-means clustering method to cluster all selected participants’ vectors, with the optimal number of clusters determined by the elbow method^57^. To achieve robust and stable clustering results, the clustering process was executed 500 times with different centroid seeds, and the results with the lowest within-cluster sum of squares were selected for the final analysis. After clustering, we conducted a comparative analysis of participants’ socio-demographics and PHQ-8 scores across the identified clusters using the Kruskal-Wallis (KW) test^58^. Additionally, for each identified cluster, we reported the seasonal changes in physical activity levels (measured in daily step count). Furthermore, the clustering analysis was also separately performed on each study site.

### Multilevel mediation analysis—exploring interplays between weather conditions, physical activity, and depression severity

**Fig. 1:**
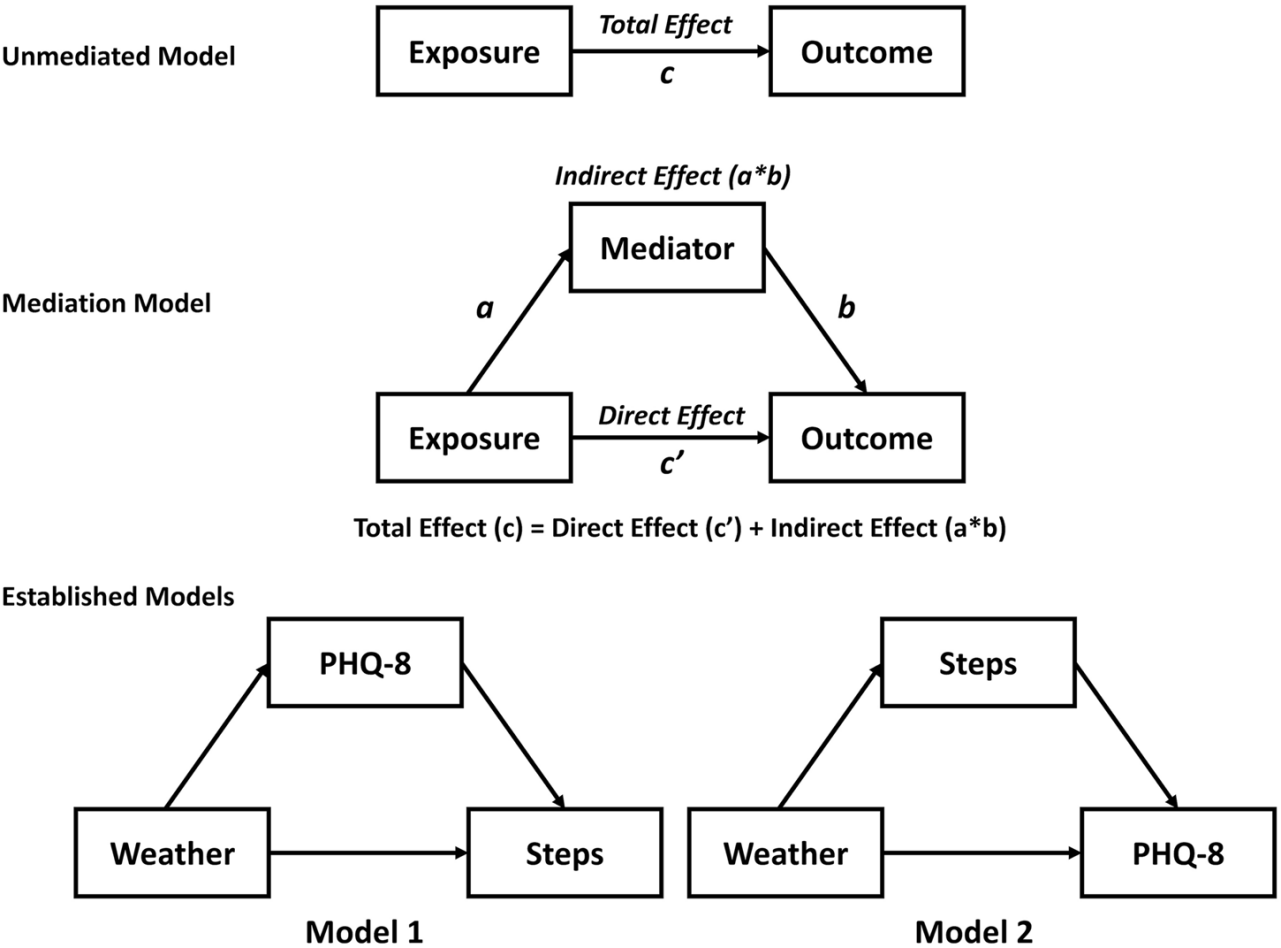
Schematic representation of unmediated and mediated models along with the implementations of mediated pathway analysis in the present study. The mediation model decomposes the total exposure-outcome effect (c path) into a direct effect (c’ path) and an indirect effect via a mediator, where the indirect effect is calculated by multiplying the effect of exposure on the mediator (a path) by the effect of the mediator on the outcome (b path). In this paper, each weather variable serves as the exposure, with one model (Model 1) considering depression symptom severity (PHQ-8) as the mediator and physical activity (Step Count) as the outcome, and another (Model 2) reversing these roles.

Mediation analysis is a statistical approach used for understanding the mechanisms underlying the exposure-outcome relationship by partitioning the total effect into direct and indirect components through an intervening variable, commonly referred to as a mediator^59^. Since our data are longitudinal (each participant has multiple measurements), we employed the 1-1-1 multilevel mediation model, in which all measures were collected at the individual level^60^. This model estimates the following effects: (1) total effect—the exposure-outcome effect without considering the effect of the mediator (c path), (2) direct effect—the effect of the exposure on the outcome when adjusted for the mediator (c’ path), (3) indirect effect—the effect of the exposure on the outcome through the mediator (a*b), (4) the effect of exposure on the mediator (a path), and (5) the effect of the mediator on the outcome (b path) (Fig. 1).

We defined each weather measure as an exposure variable and constructed two distinct mediation models for each weather variable. The first model (Model 1) considered depression symptom severity (PHQ-8) as the mediator and physical activity (Step Count) as the outcome, while the second (Model 2) treated physical activity as the mediator and depression severity as the outcome variable. The covariates mentioned above (see “Measures” section) were included to mitigate the risk of biased effect estimations^59^.

### Subgroup analysis for distinct affective responses to varying weather conditions

We hypothesized that the presence of different affective responses to varying weather conditions within a population may obscure the correlation between weather conditions and depression at the cohort level. Similarly, mediating effects that may be significant within specific weather response subgroups could become obscured or attenuated when analyzed across the entire cohort. To identify distinct subgroups, we calculated the Spearman correlation coefficients between each participant’s PHQ-8 scores and each weather variable. Following the guidelines for interpreting correlation coefficients^61^, we set ±0.2 as the threshold for weak correlation, which is suitable for our current exploratory analysis with the limited sample size to sensitively detect potential associations warranting future in-depth investigation. Accordingly, participants were categorized into “Positive-Correlation” (Spearman Coefficient >0.2), “Negative-Correlation” (Spearman Coefficient < −0.2), and “No-Correlation” (−0.2 ≤ Spearman Coefficient ≤ 0.2) subgroups, indicating different affective responses to weather conditions. For instance, participants in the “Positive-Correlation” subgroup for temperature have PHQ-8 scores that positively correlate with temperature, indicating that higher temperatures are associated with more severe symptoms. Subsequently, the mediation model was applied independently to the three subgroups for each weather variable.

## Results

### Data overview

We analyzed 12,490 PHQ-8 questionnaires with corresponding weather information and Fitbit step count recordings from 428 participants per the data inclusion criteria (see “Methods”). The selected cohort had a median age of 50.0 years (IQR: 32.0–60.0) and was predominantly female (*N* = 331, 77.3%), with a median PHQ-8 score of 9.5 (IQR: 6.0–13.7). The median of individual average daily steps for study participants was 7135.5 (IQR: 4967.9–9267.3). Participants’ socio-demographics (except for gender) and PHQ-8 score distributions were significantly different across the three study sites. Participants from CIBER (Spain) were the oldest and had the highest PHQ-8 scores. Further detailed socio-demographic information is provided in Table 1. Additionally, a sensitivity analysis showed no significant socio-demographic differences between the selected cohort in this study and the full RADAR-MDD study cohort. See Supplementary Table 1 for additional details.

**Table 1:** A summary of characteristics of participants in the selected cohort in this study, with comparisons across three study sites using Kruskal–Wallis tests

Characteristics	Overall	CIBER (Spain)	KCL (United Kingdoms)	VUMC (Netherlands)	*p* value
Number of participants	428	90	251	87	
All PHQ8, median [IQR]	9.5 [6.0,13.7]	13.4 [8.6,17.5]	8.9 [5.5,12.5]	9.1 [6.1,11.9]	<0.001
Baseline PHQ8, median [IQR]	10.0 [6.0,15.0]	14.5 [10.0,18.8]	9.0 [6.0,13.0]	8.0 [6.0,13.0]	<0.001
Age, median [IQR]	50.0 [32.0,60.0]	55.0 [45.5,61.0]	47.0 [31.0,59.0]	40.0 [26.5,58.5]	<0.001
Years in education, median [IQR]	16.0 [13.0,19.0]	11.0 [9.0,16.0]	17.0 [14.0,19.0]	17.0 [14.0,20.5]	<0.001
Female, *n* (%)	331 (77.3)	65 (72.2)	195 (77.7)	71 (81.6)	0.322
Employed, *n* (%)	186 (43.5)	22 (24.4)	136 (54.2)	28 (32.2)	<0.001
Has children, *n* (%)	211 (49.3)	69 (76.7)	113 (45.0)	29 (33.3)	<0.001
Married status, *n* (%)					0.003
Single	226 (52.8)	36 (40.0)	133 (53.0)	57 (65.5)	
Married	202 (47.2)	54 (60.0)	118 (47.0)	30 (34.5)	
Annual income (£/€), *n* (%)					<0.001
<15,000	101 (23.6)	28 (31.1)	51 (20.3)	22 (25.3)	
15,000–55,000	246 (57.5)	57 (63.3)	150 (59.8)	39 (44.8)	
>55,000	70 (16.4)	5 (5.6)	50 (19.9)	15 (17.2)	

**Fig. 2:**
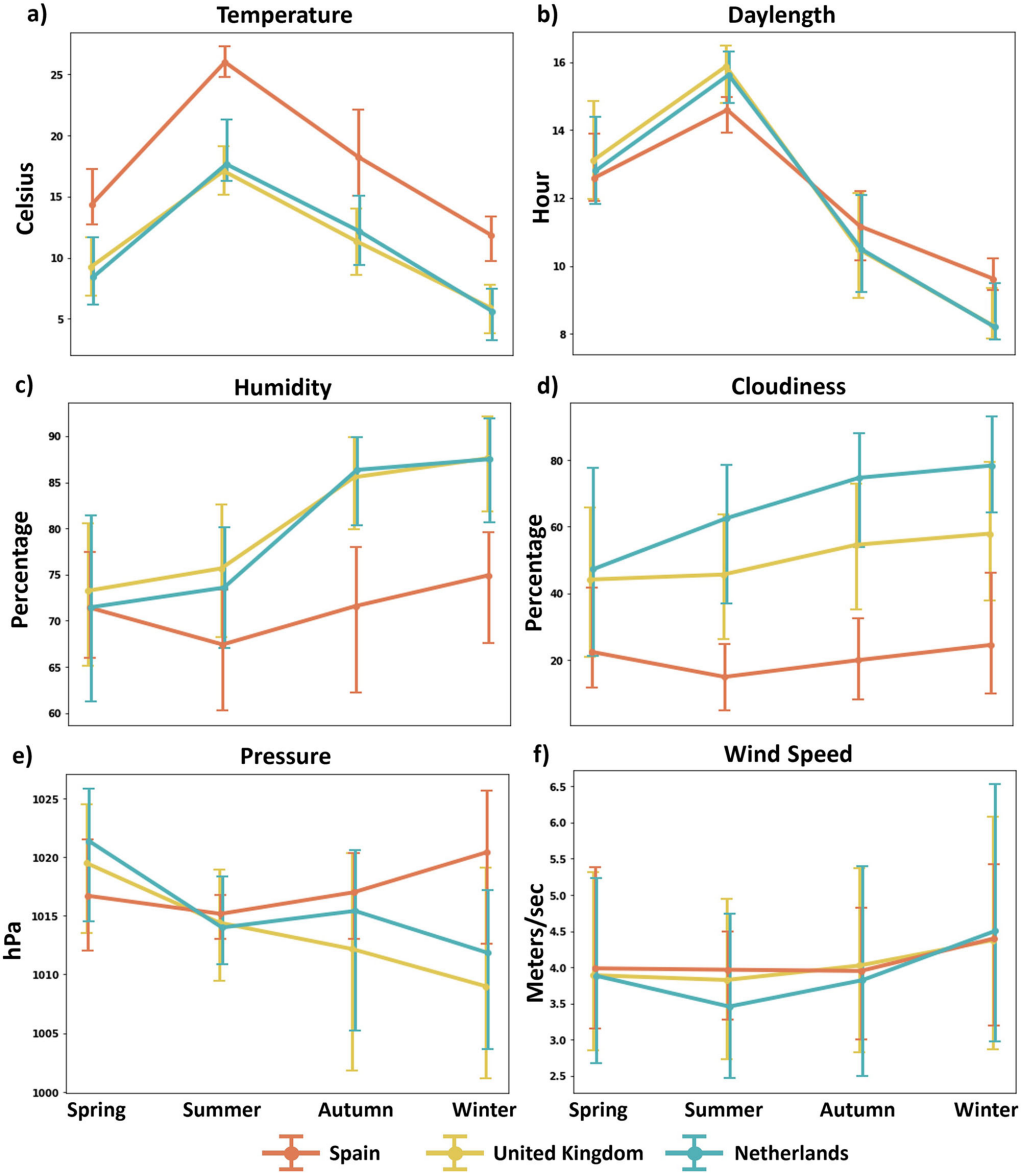
Seasonal variations in weather conditions across three study sites. Weather conditions collected in this study are **a** ambient temperature (in Celsius), **b** day length (the time between sunrise and sunset, measured in hours), **c** humidity (%), **d** cloudiness (%), **e** atmospheric pressure (in hPa), and **f** wind speed (in meters/s).

Figure 2 illustrates the seasonal fluctuations in weather variables across the three countries. The data reveal distinct seasonal patterns in ambient temperature and day length, peaking in summer and reaching the lowest values in winter. Spain exhibits higher temperatures than the UK and the Netherlands. The UK and the Netherlands experienced higher humidity and cloudiness during autumn and winter, compared to spring and summer. Conversely, Spain experienced minimal seasonal variations in humidity and cloudiness, with slightly lower values in summer and generally lower levels compared to the other countries. Furthermore, atmospheric pressure and wind speed are relatively stable across seasons, with minor variations.

### Depression severity variations across the seasons in 1 year of observation

**Fig. 3:**
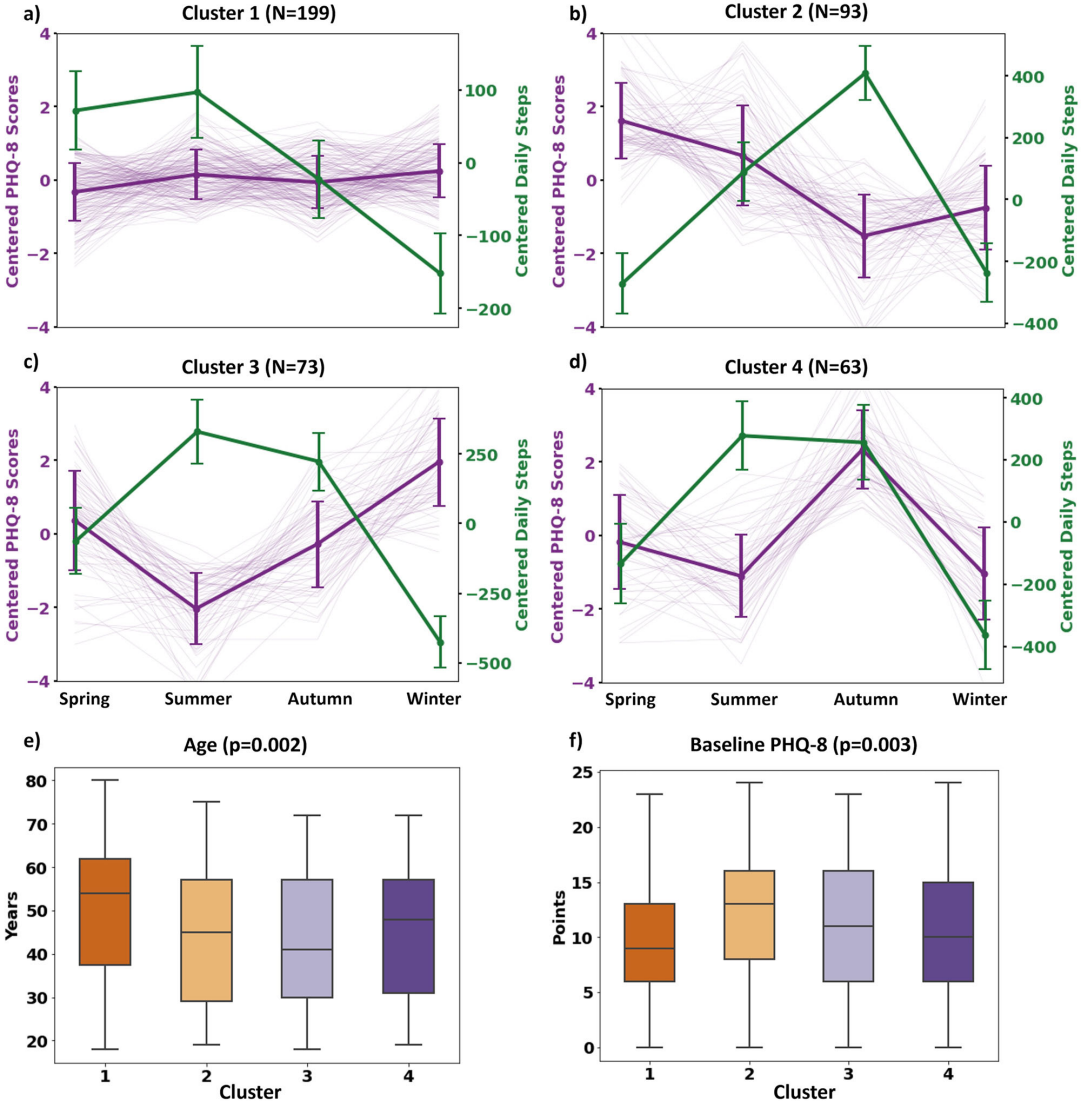
Seasonal variations in depression severity and physical activity. Four distinct patterns of seasonal variations in depression symptom severity (mean-centered PHQ-8 scores; depicted in purple) and corresponding changes in physical activity levels (mean-centered daily steps; depicted in green) within the whole cohort (**a**–**d**). Age and baseline PHQ-8 scores are significantly (Kruskal–Wallis tests) different across the four clusters (**e**, **f**). The comparisons of other socio-demographics across clusters are shown in Supplementary Table 2.

The unsupervised clustering of mean-centered PHQ-8 data revealed four distinct patterns of depression variations across seasons in the 1-year observational period among included participants. Figure 3 illustrates the changes in mean-centered PHQ-8 scores across the seasons for participants in Clusters 1–4, as well as corresponding changes in physical activity levels (measured in daily steps). The centered PHQ-8 scores represent deviations from each participant’s mean PHQ-8 score. In Cluster 1 (*N* = 199; 46.5%), participants exhibited minimal variation in their PHQ-8 scores, with only an average difference of 0.99 points between the highest and lowest scores across all seasons. Cluster 2 (*N* = 93; 21.7%) displayed the highest PHQ-8 scores in spring (1.89 points above the mean), and the lowest in autumn (1.73 points below the mean). For Cluster 3 (*N* = 73; 17.1%), participants experienced their peak PHQ-8 scores in winter (2.21 points above the mean), and the lowest in summer (2.13 points below the mean). Lastly, participants in Cluster 4 (*N* = 63; 14.7%) showed the highest PHQ-8 scores in autumn (2.64 points above the mean), and the lowest in winter (1.43 points below the mean).

In addition, participants’ baseline PHQ-8 scores and characteristics were significantly different across the four identified clusters. The baseline PHQ-8 was lowest in Cluster 1 (median: 9.0 [IQR: 6.0–13.0]), and highest in Cluster 2 (median: 13.0 [IQR: 8.0–16.0]) (KW test: *p* = 0.002) (Fig. 3f). Participants in Cluster 1 were the oldest (median age: 54.0 years [IQR: 37.5–62.0]), while those in Cluster 3 were the youngest (median age: 41.0 years [IQR: 30.0–57.0]) (KW test: *p* = 0.002) (Fig. 3e). The proportion of female participants was lower in Cluster 1 (70.9%) compared to other clusters (Cluster 2: 83.9%, Cluster 3: 86.3%, and Cluster 4: 77.8%) (KW test: *p* = 0.02). Additionally, the proportion of participants from each study site significantly varied across the clusters (KW test: *p* = 0.02). Details of the comparative analysis across the clusters are provided in Supplementary Table 2.

Additionally, in Cluster 1, where the PHQ-8 variation across the seasons is the smallest, the physical activity pattern appears to follow a seasonal trend, peaking in summer and declining to its lowest point in winter.

The clustering results at each study site are summarized in Supplementary Figs. 2–4. While similar seasonal patterns of depression severity were also observed at each site, the numbers of participants in some clusters at the Spain and the Netherlands study sites were relatively small and should be interpreted with caution.

### Mediating effects of weather conditions on physical activity and depression severity

**Fig. 4:**
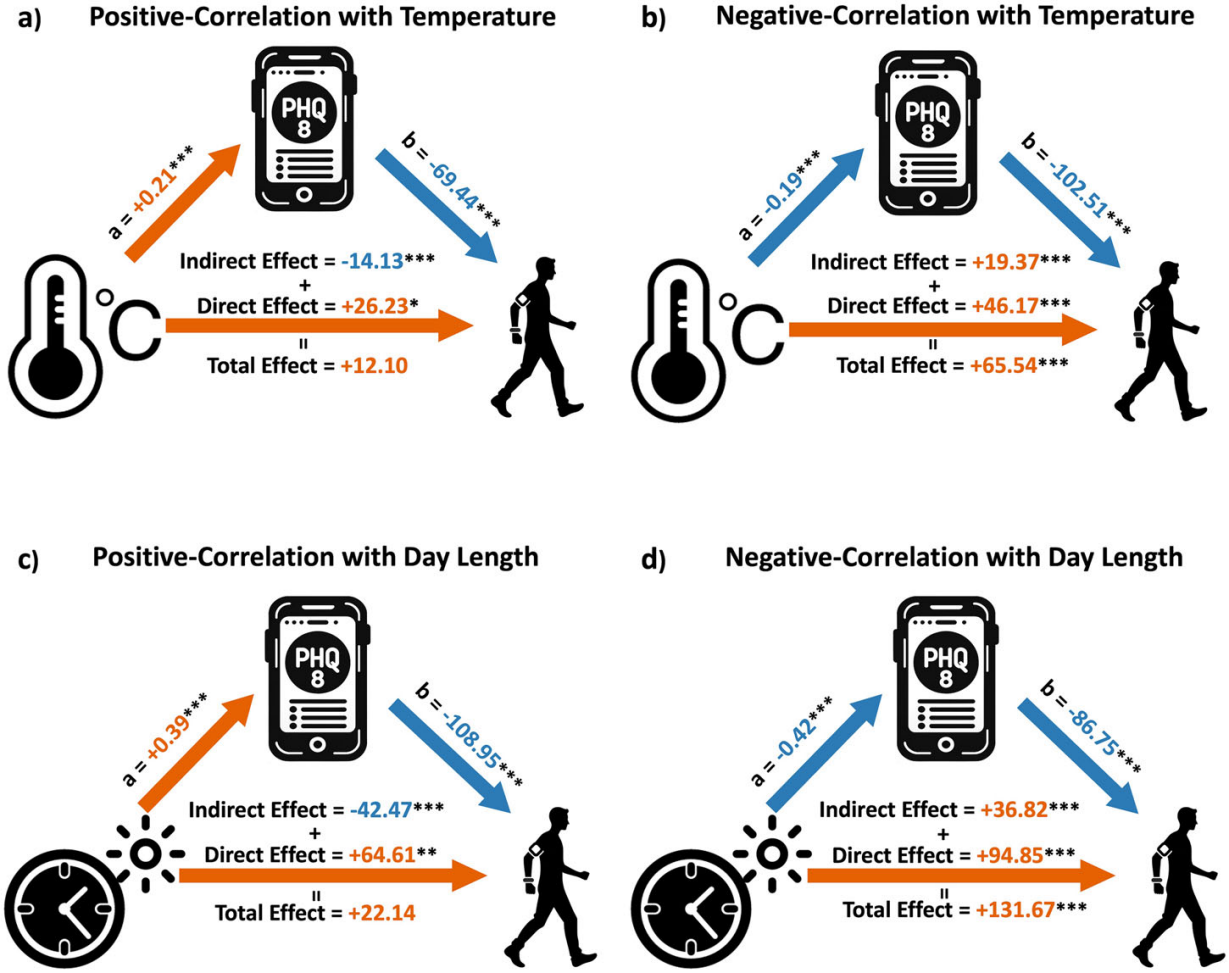
The path diagrams and effects of mediation models for Positive-Correlation and Negative-Correlation subgroups for temperature and day length. **a** Positive-Correlation subgroup for temperature, **b** Negative-Correlation subgroup for temperature, **c** Positive-Correlation subgroup for day length, and **d** Negative-Correlation subgroup for day length. The subgroups were assigned based on Spearman Coefficients between depression severity and weather conditions (see “Methods”). The orange indicates the positive effect while blue represents the negative effect. The significance levels: *p < 0.05, **p < 0.01, ***p < 0.001.

While the indirect effects of weather changes on physical activity via depression severity (Model 1) were modest in the entire study cohort, these impacts, especially for temperature and day length, became relatively substantial in subgroups (Table 2). Specifically, to test the mediating effects among participants with different affective responses to weather conditions, participants were assigned to three subgroups based on individual Spearman coefficients between depression severity (PHQ-8) and each weather variable (see “Methods”). Temperature and day length significantly affected depression severity, which in turn influenced the physical activity in the Positive-Correlation and Negative-Correlation subgroups, and these effects were opposite (Fig. 4 and Table 2). Detailed results of these two models are reported below.

**Table 2:** Outcomes of mediation models assessing the effect of weather conditions on physical activity via depression severity (PHQ-8) across the entire cohort and subgroups (Positive-Correlation, Negative-Correlation, and No-Correlation; see “Methods”)

	Temperature (Celsius)	Daylength (h)	Humidity (%)	Cloudiness (%)	Pressure (hPa)	Wind speed (meters/s)
Entire cohort						
Total effects (c)	46.09***	65.6***	−7.25**	−2.71*	−1.68	−0.69
Direct effect (c’)	44.08***	61.91***	−6.53**	−2.47*	−2.65	1.96
Indirect effect (a*b)	2.01***	3.69**	−0.73*	−0.24	0.97**	−2.66
a path	−0.02**	−0.04**	0.01*	0.003	−0.01**	0.03
b path	−87.84***	−89.01***	−90.22***	−90.36***	−91.08***	−90.8***
Positive-Correlation subgroup						
Total effects (c)	12.1	22.14	−22.78***	−8.61***	4.98	−57.58
Direct effect (c’)	26.23*	64.61**	−12.2*	−5.8**	14.15	−37.56
Indirect effect (a*b)	−14.13***	−42.47***	−10.58***	−2.81***	−9.17***	−20.03*
a path	0.21***	0.39***	0.09***	0.04***	0.08***	0.47***
b path	−69.44***	−108.95***	−112.22***	−75.74***	−119.04***	−43.47*
Negative-Correlation subgroup						
Total effects (c)	65.54***	131.67***	−2.63	1.31	12.17*	107.96***
Direct effect (c’)	46.17***	94.85***	−8.16	−1.52	2.73	85.29**
Indirect effect (a*b)	19.37***	36.82***	5.53***	2.83***	9.44***	22.66***
a path	−0.19***	−0.42***	−0.1***	−0.04***	−0.09***	−0.39***
b path	−102.51***	−86.75***	−60.17***	−67.93***	−106.49***	−57.27**
No-Correlation subgroup						
Total effects (c)	47.04***	44.08***	−1.18	−1.6	−7.06*	−10.37
Direct effect (c’)	47.1***	43.53***	−0.88	−1.39	−7.4*	−10.0
Indirect effect (a*b)	−0.06	0.56	−0.31	−0.22	0.34	−0.37
a path	0.001	−0.01	0.003	0.002	−0.004	0.01
b path	−80.24***	−75.38***	−87.6***	−103.08***	−81.73***	−111.08***

Significance levels: *p < 0.05; **p < 0.01; ***p < 0.001.

#### Temperature

For each 10 °C increase, divergent effects on PHQ-8 scores were observed across subgroups: a 2.1-point increase in the Positive-Correlation subgroup (*p* < 0.001), a 1.9-point decrease in the Negative-Correlation subgroup (*p* < 0.001), and no significant change in the No-Correlation subgroup. Due to different responses to temperature variations (a path) and the inverse relationship between depression severity and physical activity (b path), a 10 °C increase in temperature led to opposite indirect effects on daily step counts through depression severity: a decrease of 141.3 steps in the Positive-Correlation subgroup and an increase of 193.7 steps in the Negative-Correlation subgroup (*p* < 0.001), and modest effects (0.6 steps; *p* = 0.94) in No-Correlation subgroup. The direct effects of temperature on physical activity were significant and positive across all subgroups (increments of 262.3 steps (*p* = 0.02), 461.7 steps (*p* < 0.001), and 471.0 steps (*p* < 0.001) per 10 °C rise in Positive-Correlation, Negative-Correlation, and No-Correlation subgroups, respectively). Thus, the total effect, integrating direct and indirect influences, showed an increase of 262.3 − 141.3 = 121.0 steps in the Positive-Correlation subgroup (not significant), 461.7 + 193.7 = 655.4 steps in the Negative-Correlation subgroup (*p* < 0.001), and 471.0 − 0.6 = 470.4 steps in the No-Correlation subgroup (*p* < 0.001) per 10 °C rise.

#### Day length

Observations across subgroups revealed that each additional hour of day length resulted in varying PHQ-8 score changes: a 0.39-point increase in the Positive-Correlation subgroup (*p* < 0.001), a 0.42-point decrease in the Negative-Correlation subgroup (*p* < 0.001), and insignificant changes in the No-Correlation subgroup. Similarly, for the indirect effects, an extra hour of day length contributed to contrasting daily step count outcomes: a reduction of 42.47 steps in the Positive-Correlation subgroup and an increment of 36.82 steps in the Negative-Correlation subgroup (*p* < 0.001). The direct influence of day length on physical activity was also uniformly positive and significant across subgroups (each hour increment led to increases of 64.61 (*p* = 0.008), 94.85 (*p* < 0.001), and 43.53 (*p* < 0.001) daily steps in Positive-Correlation, Negative-Correlation, and No-Correlation subgroups, respectively). Consequently, the total effect demonstrated step count increment of 22.14 steps (not significant) in the Positive-Correlation subgroup, 131.67 steps (*p* < 0.001) in the Negative-Correlation subgroup, and 44.08 steps (*p* < 0.001) in the No-Correlation subgroup per additional hour of day length.

Furthermore, for other weather variables such as humidity, cloudiness, pressure, and wind speed, the mediating effects on physical activity and their impacts on depression severity were relatively modest (Table 2), considering the annual variability range of these variables (Fig. 2). On the other hand, the examination of how weather conditions affect depression severity through physical activity (Model 2) found the indirect effects were modest, indicating the impact of weather on depression is predominantly direct. Detailed results of Model 2 are available in Supplementary Table 3.

## Discussion

Our analysis of long-term data collected from a clinically depressed population in real-world settings demonstrated complex interplays between weather conditions, physical activity, and depression severity. Specifically, changes in weather conditions, such as temperature and day length, significantly influenced depression severity, which in turn impacted participants’ physical activity levels. Notably, these indirect influences manifested differently or even oppositely across participants with varying responses to weather. Additionally, our findings showed distinct patterns of depression severity variations across the seasons in the 1-year observational period in the present cohort. Although these findings require further validation through future multi-year longitudinal studies, they contribute to the hypothesis of the existence of different types of SAD^35,36^ and diverse responses to weather^33^ within a population.

Unsupervised clustering analysis showed distinct subgroups of participants with differences in depression severity variations across seasons in the study cohort, offering potential data-driven explanations for previous inconsistencies reported in the literature concerning seasonality’s effects on depression^28,62^. Notably, one cluster with a considerable portion of participants (Cluster 1 in Fig. 3) exhibited minimal seasonal variations, suggesting that seasonal influences might affect specific subgroups, potentially clarifying why studies with broader populations often report modest or negligible effects of seasonality^23,27,28^. Furthermore, our analysis revealed that older participants typically showed minimal seasonal variations, which is consistent with findings from a meta-analysis of 20 population studies indicating that SAD is more prevalent among younger adults^20^. Additionally, our data show a higher prevalence of pronounced seasonal fluctuations among females (Clusters 2 and 3 in Fig. 3), aligning with prior research indicating a stronger seasonal impact on depression in females^20,23,63^. However, while our analysis of the study population indicated the distinct patterns of depression variations across the seasons in 1 year, it is important to note that other personal life events (e.g., exams, workload, divorce, and bereavement) that were not assessed as part of the study could also influence participants’ fluctuations in depression severity. Moreover, these findings were limited to 1 year of observation. Therefore, there is a need to validate our findings in future longitudinal studies covering multiple years.

Mediation analysis further decomposed the overall impact of weather on physical activity into direct effects caused by the weather itself and indirect effects resulting from weather-induced changes in depression severity and its downstream effect on physical activity. Our results showed that temperature and day length significantly influenced depression severity, which, in turn, indirectly impacted physical activity levels. However, these indirect effects were reversed between subgroups with opposite responses (Positive-Correlation and Negative-Correlation subgroups) to these two weather variables. Specifically, for participants whose depression severity decreased with rising temperatures (Negative-Correlation), the direct impact of temperature and its indirect effect through reduced depression had the same direction, resulting in a more pronounced overall effect on physical activity. In contrast, for the Positive-Correlation subgroup, rising temperatures led to increased depression severity, which subsequently caused a decrease in physical activity. As a result, these opposing direct and indirect effects led to an overall combined effect that was insignificant for physical activity levels (Fig. 4).

In Europe, temperature and day length exhibit substantial variations throughout the year, although these changes differ across latitudes. In our dataset, the median annual temperature fluctuation was 19.6 °C (IQR: 16.2–23.2), and the median variation in day length was 8.5 h (IQR: 6.0–8.8). Consequently, mediation models (Fig. 4) showed annual temperature fluctuations could result in an average increase of 4.1 points (0.21*19.6) in PHQ-8 for the Positive-Correlation subgroup and reduce PHQ-8 average by −3.7 points (−0.19*19.6) for the Negative-Correlation subgroup while also impacting physical activity by total changes of 237.2 and 1284.6 steps respectively (direct effects were 514.1 and 904.9 steps). Similarly, annual changes in day length led to a PHQ-8 score change of +3.3 and −3.6 points and impacted physical activity with total effects of 188.2 and 1119.2 steps for the Positive-Correlation and Negative-Correlation subgroup respectively (direct effects were 549.2 and 806.2 steps respectively).

Previous studies show that a 3-point change in the PHQ-9 is clinically significant^64,65^. In that context, a change of 3.3-4.1 points observed in this study in the PHQ-8, comprising 8 of the 9 items of PHQ-9, can be considered clinically meaningful. Prior health research using physical mobility data gathered from wearable devices has demonstrated that a change in daily step count of 500 steps can be clinically meaningful^66–68^. Therefore, considering magnitudes of changes in depression severity and step count linked to changes in temperature and day length across subgroups of participants with varying responses to weather may help inform personalized assessment of depression symptoms in clinical trials and for optimizing interventions.

Prior studies have also reported heterogeneity in people’s affective responses to temperature and day length. Klimstra et al. reported that among 497 participants over 30 days, 16.8% experienced mood improvements with higher temperatures and more sunshine, while 26.8% reacted negatively, and 47.8% remained unaffected^33^. Other studies have noted conflicting impacts of temperature on mental health: some find it may alleviate depression symptoms^21^, whereas others link higher temperatures to increased mental health disorders and suicide risks^69^. Regarding day length, certain studies have identified an improvement in depression symptoms with increased daylight^70^, supporting the effectiveness of light therapy in treating depression^71^. Conversely, other research has reported poorer sleep and mood in some individuals during summer, potentially linked to summer SAD^72,73^.

Previous studies have demonstrated the efficacy of diverse interventions, such as exercise, light therapy, and antidepressants, in preventing depression; however, their effectiveness varies significantly across individuals^43,71,74–76^. Contextualizing the impact of seasonal depression variations can help identify factors influencing the effectiveness of these interventions, enabling healthcare research and real-world practice substantially. In clinical studies, for instance, stratifying populations based on the impact of weather or SAD could help deconvolve the heterogeneity of treatment responses across treatment and placebo groups. Additionally, recognizing different types of SAD allows healthcare providers to implement preventive measures, such as adjusting medication dosages or intensifying psychotherapy, before or during high-risk periods^74,77^. Moreover, understanding a patient’s response to changes in weather can enhance personalized intervention strategies. For example, recommending cooler, shaded areas for exercise intervention for those whose depression worsens with rising temperatures, or warmer environments for those sensitive to cold. However, there is a need for future qualitative research to work with participants experiencing SAD to understand how weather changes affect their daily functioning. Further research is also needed to improve the effectiveness of personalized interventions based on individuals’ responses to seasonal changes in real-world applications.

Our findings should be interpreted within the context of several limitations. First, our cohort, which consists predominantly of females with a history of depression and is based in Europe, may limit our generalizability to more diverse or non-depressed populations. Second, while the present study analyzed long-term data, the potential patterns of seasonal variation in depression were observed in a single year. There is a need for multi-year longitudinal studies to further understand and validate the individualized variation over multiple years and seasons. Third, over half of our data were collected during the COVID-19 pandemic, which may have introduced biases despite including a covariate of national lockdown; thus, our findings need to be further assessed in post-pandemic datasets. Fourth, the weather data used in this study were retrospectively obtained based on the city information provided at the time of PHQ-8 submission. This approach only allowed us to capture the weather conditions on the day of submission, lacking dynamic information during the period before the PHQ-8 assessment. Future digital studies might benefit from concurrently collecting passive data and weather information, providing a richer and more dynamic context for analysis. Fifth, since the numbers of participants in some clusters at the Spain and the Netherlands study sites were relatively small, the results of separate site-specific cluster analyses should be interpreted with caution, highlighting the need for future larger research to explore seasonal patterns between countries. Sixth, information on whether study participants were long-term or temporary residents was not collected. Future large-scale longitudinal studies should assess potential differences in weather tolerance between long-term residents and those who have recently relocated and may be temporary residents.

In conclusion, this study further contributes to the hypothesis of heterogeneity in individuals’ depression variations across seasons and responses to weather within populations. Our findings enhance the understanding of complex mechanisms of how external weather changes could affect individuals’ depression severity and physical activity. Furthermore, we discussed that stratifying populations based on the impact of weather or SAD may help disentangle the heterogeneity of treatment responses in clinical research, as well as the need for personalized approaches in managing depression based on different individual responses to weather changes. Future research is needed to validate these findings over longer periods and across more diverse populations.

## Supplementary information

Supplementary

## Data Availability

The processed and anonymized data used for the present study can be made available through reasonable requests to the RADAR-CNS consortium, but the raw passive data and demographics cannot be made available due to participant safety and data privacy issues. Please email the corresponding author for details.

## Code availability

The code for the analysis is available through a GitHub code repository (https://github.com/kcl-bhi/RADAR_MDD_Weather_Analysis).
