# Supplementary material for: Assessing seasonal and weather effects on depression and physical activity using mobile health data
Source: Npj Ment Health Res. 2025 Apr 18;4:11. doi: 10.1038/s44184-025-00125-x (PMC12008285; doi:10.1038/s44184-025-00125-x)

**Supplementary Table 1. A summary of socio-demographics of all participants in the RADAR-MDD, selected participants in this paper, and excluded participants, with comparisons using the Kruskal-Wallis tests.**

| **Characteristics** | **All participants in RADAR-MDD** | **Selected participants in this paper** | **Excluded participants** | **p value** |
| --- | --- | --- | --- | --- |
| **Number of participants** | 623 | 428 | 195 |  |
| **Age, median [IQR]** | 49.0 [32.0,59.0] | 50.0 [32.0,60.0] | 49.0 [33.5,56.5] | 0.336 |
| **Years in education, median [IQR]** | 16.0 [13.0,19.0] | 16.0 [13.0,19.0] | 15.0 [13.0,18.0] | 0.358 |
| **Female, n (%)** | 471 (75.6) | 331 (77.3) | 140 (71.8) | 0.164 |
| **Employed, n (%)** | 260 (41.7) | 186 (43.5) | 74 (37.9) | 0.167 |
| **Has children, n (%)** | 311 (50.0) | 211 (49.3) | 100 (51.5) | 0.665 |
| **Married Status, n (%)** |  |  |  | 0.877 |
| **Single** | 332 (53.3) | 226 (52.8) | 106 (54.4) |  |
| **Married** | 291 (46.7) | 202 (47.2) | 89 (45.6) |  |
| **Annual income (£/€), n (%)** |  |  |  | 0.871 |
| **< 15,000** | 154 (24.7) | 101 (23.6) | 53 (27.2) |  |
| **15,000-55,000** | 354 (56.8) | 246 (57.5) | 108 (55.4) |  |
| **> 55,000** | 98 (15.7) | 70 (16.4) | 29 (14.9) |  |
| **Site, n (%)** |  |  |  | 0.004 |
| **CIBER (Spain)** | 155 (24.9) | 90 (21.0) | 65 (33.3) |  |
| **KCL (UK)** | 350 (56.2) | 251 (58.6) | 99 (50.8) |  |
| **VUMC (Netherlands)** | 118 (18.9) | 87 (20.3) | 31 (15.9) |  |

**Supplementary Table 2. Summary of socio-demographics and PHQ-8 scores across four distinct seasonal variations in depression severity, with the comparisons using the Kruskal-Wallis tests.**

| **Characteristics** | **Cluster 1** | **Cluster 2** | **Cluster 3** | **Cluster 4** | **P value** |
| --- | --- | --- | --- | --- | --- |
| **Number of participants** | 199 | 93 | 73 | 63 |  |
| **All PHQ8, median [IQR]** | 8.6 [4.9,12.2] | 10.8 [7.6,15.2] | 9.7 [7.0,14.4] | 10.7 [7.9,13.9] | 0.002 |
| **Baseline PHQ8, median [IQR]** | 9.0 [6.0,13.0] | 13.0 [8.0,16.0] | 11.0 [6.0,16.0] | 10.0 [6.0,15.0] | 0.003 |
| **Age, median [IQR]** | 54.0 [37.5,62.0] | 45.0 [29.0,57.0] | 41.0 [30.0,57.0] | 48.0 [31.0,57.0] | 0.002 |
| **Years in education, median [IQR]** | 16.0 [13.0,19.0] | 17.0 [12.0,20.0] | 15.0 [13.0,18.0] | 15.0 [12.5,18.5] | 0.731 |
| **Female, n (%)** | 141 (70.9) | 78 (83.9) | 63 (86.3) | 49 (77.8) | 0.016 |
| **Employed, n (%)** | 84 (42.2) | 47 (50.5) | 32 (43.8) | 23 (36.5) | 0.250 |
| **Has children, n (%)** | 107 (53.8) | 40 (43.0) | 33 (45.2) | 31 (49.2) | 0.314 |
| **Married Status, n (%)** |  |  |  |  | 0.332 |
| **Single** | 97 (48.7) | 53 (57.0) | 38 (52.1) | 38 (60.3) |  |
| **Married** | 102 (51.3) | 40 (43.0) | 35 (47.9) | 25 (39.7) |  |
| **Annual income (£/€), n (%)** |  |  |  |  | 0.182 |
| **< 15,000** | 48 (24.1) | 18 (19.4) | 21 (28.8) | 14 (22.2) |  |
| **15,000-55,000** | 111 (55.8) | 57 (61.3) | 37 (50.7) | 41 (65.1) |  |
| **> 55,000** | 37 (18.6) | 15 (16.1) | 12 (16.4) | 6 (9.5) |  |
| **Site, n (%)** |  |  |  |  | 0.020 |
| **CIBER (Spain)** | 38 (19.1) | 27 (29.0) | 10 (13.7) | 15 (23.8) |  |
| **KCL (UK)** | 129 (64.8) | 50 (53.8) | 41 (56.2) | 31 (49.2) |  |
| **VUMC (Netherlands)** | 32 (16.1) | 16 (17.2) | 22 (30.1) | 17 (27.0) |  |

**Supplementary Table 3. Outcomes of mediation models assessing the effect of weather conditions on depression severity via physical activity (Model 2 in Figure 1) across the entire cohort and subgroups (Positive Reactivity, Negative Reactivity, and Unaffected; See Methods). Note, to facilitate the display of coefficients, the step counts have been divided by 1000. Significance Levels: *p<0.05, **p<0.01, ***p<0.001.**

|  | **Temperature** | **Daylength** | **Humidity** | **Cloudiness** | **Pressure** | **Wind Speed** |
| --- | --- | --- | --- | --- | --- | --- |
| **Entire Cohort** |  |  |  |  |  |  |
| Total Effects (c) | -0.02 *** | -0.04 ** | 0.01 * | 0.003 | -0.01 * | 0.03 |
| Direct Effect (c') | -0.01 | -0.03 | 0.01 | 0.002 | -0.01 ** | 0.03 |
| Indirect Effect (a*b) | -0.01 *** | -0.01 *** | 0.002 *** | 6e-04 *** | 4e-04 | 1e-04 |
| a path | 0.05 *** | 0.07 *** | -0.01 ** | -0.003 ** | -0.002 | 0 |
| b path | -0.22 *** | -0.22 *** | -0.22 *** | -0.22 *** | -0.22 *** | -0.22 *** |
| **Positive Reactivity** |  |  |  |  |  |  |
| Total Effects (c) | 0.2 *** | 0.39 *** | 0.09 *** | 0.04 *** | 0.08 *** | 0.47 *** |
| Direct Effect (c') | 0.21 *** | 0.39 *** | 0.09 *** | 0.04 *** | 0.08 *** | 0.47 *** |
| Indirect Effect (a*b) | -0.002 | -0.01 | 0.01 *** | 0.002 *** | -0.001 | 0.01 |
| a path | 0.01 | 0.02 | -0.02 *** | -0.01 *** | 0.01 | -0.06 |
| b path | -0.2 *** | -0.23 *** | -0.25 *** | -0.2 *** | -0.23 *** | -0.1 * |
| **Negative Reactivity** |  |  |  |  |  |  |
| Total Effects (c) | -0.19 *** | -0.42 *** | -0.1 *** | -0.04 *** | -0.09 *** | -0.39 *** |
| Direct Effect (c') | -0.18 *** | -0.4 *** | -0.1 *** | -0.04 *** | -0.08 *** | -0.37 *** |
| Indirect Effect (a*b) | -0.02 *** | -0.02 *** | 4e-04 | -2e-04 | -0.003 * | -0.02 *** |
| a path | 0.07 *** | 0.13 *** | -0.002 | 0.001 | 0.01 * | 0.11 ** |
| b path | -0.23 *** | -0.18 *** | -0.15 *** | -0.16 *** | -0.27 *** | -0.17 ** |
| **Unaffected** |  |  |  |  |  |  |
| Total Effects (c) | 3e-04 | -0.01 | 0.003 | 0.002 | -0.004 | 0.004 |
| Direct Effect (c') | 0.01 | 6e-04 | 0.003 | 0.002 | -0.01 | 0.002 |
| Indirect Effect (a*b) | -0.01 *** | -0.01 *** | 2e-04 | 4e-04 | 0.002 *** | 0.002 |
| a path | 0.05 *** | 0.04 ** | -0.001 | -0.002 | -0.01 * | -0.01 |
| b path | -0.17 *** | -0.19 *** | -0.2 *** | -0.23 *** | -0.2 *** | -0.25 *** |

**Supplementary Figure 1. The interplay between weather, physical activity, and depression severity. Physical activity and depression could be impacted by changes in weather through two distinct pathways: (1) weather impacts depression severity, which in turn influences physical activity (purple), and (2) weather impacts physical activity, which then affects the severity of depression (green).**


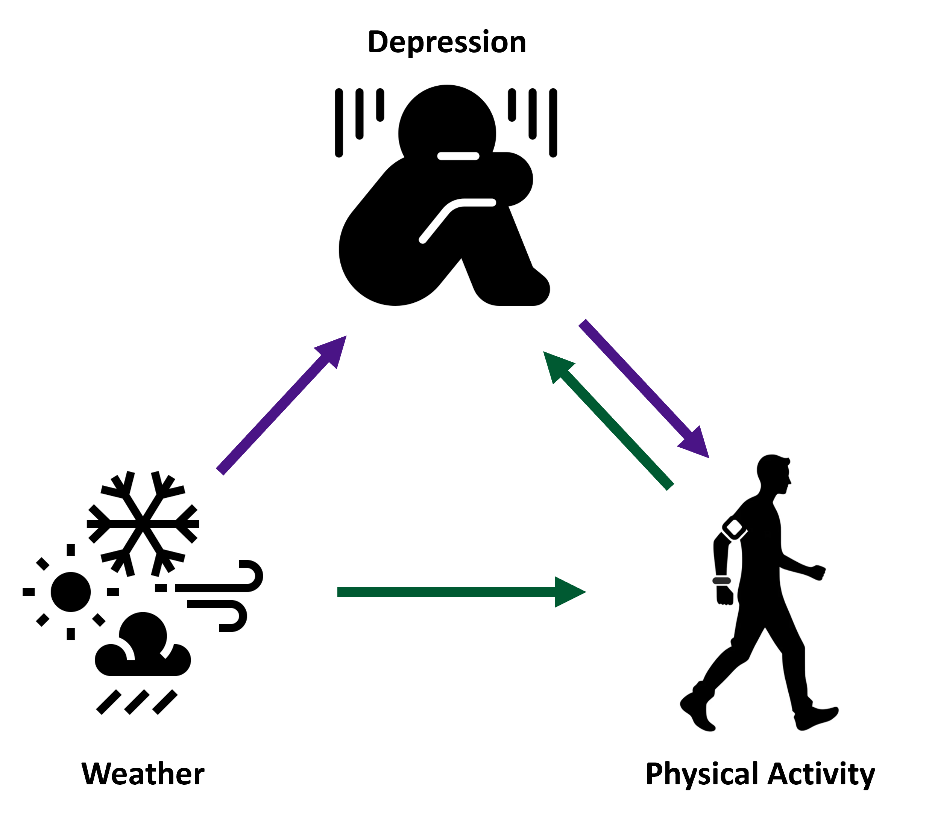


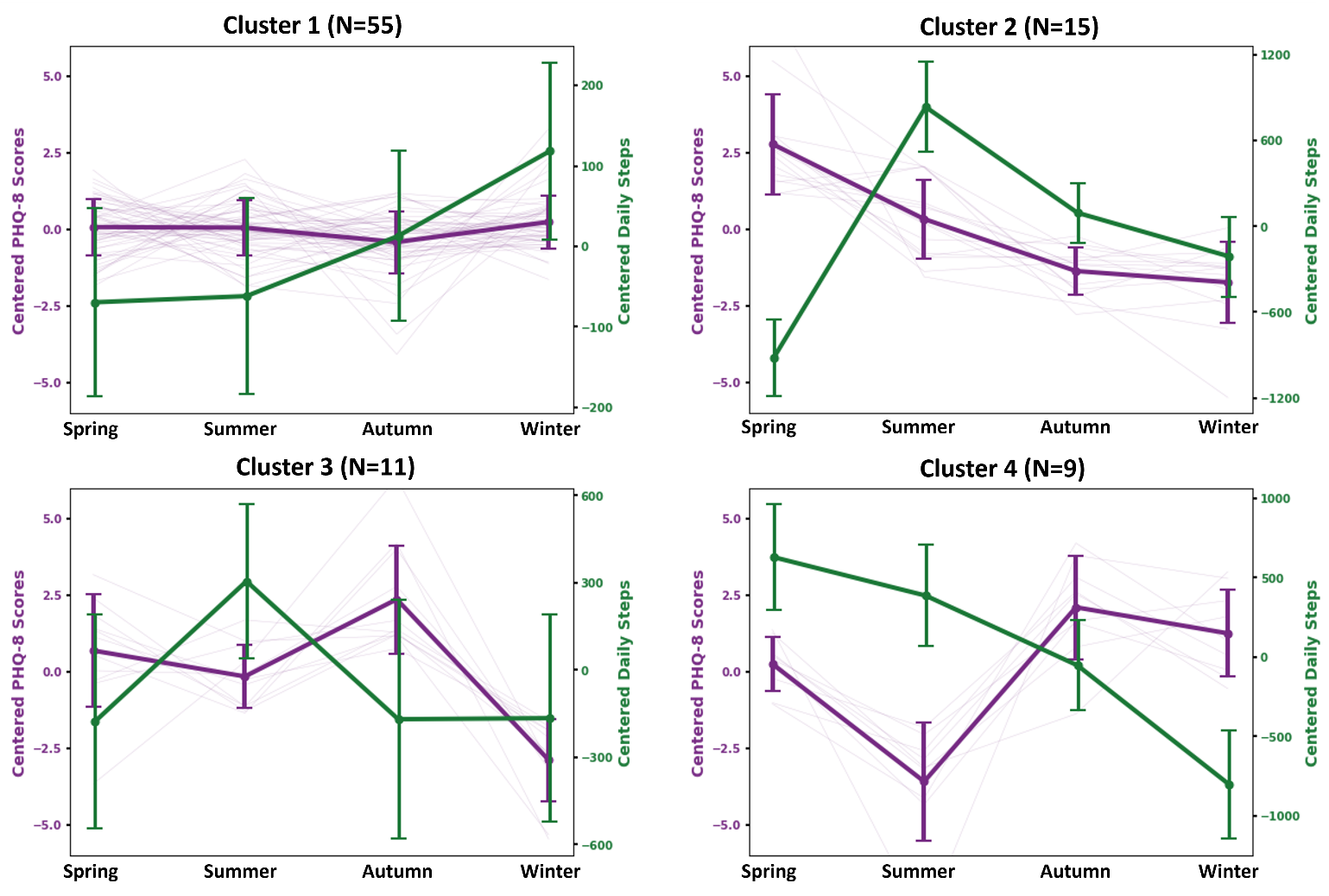
**Supplementary Figure 2. Four distinct patterns of seasonal variations in depression symptom severity (mean-centered PHQ-8 scores, depicted in purple) and corresponding changes in physical activity levels (mean-centered daily steps, depicted in green) in the CIBER study site (Spain).**

**Supplementary Figure 3. Four distinct patterns of seasonal variations in depression symptom severity (mean-centered PHQ-8 scores, depicted in purple) and corresponding changes in physical activity levels (mean-centered daily steps, depicted in green) in the KCL study site (United Kingdom).**


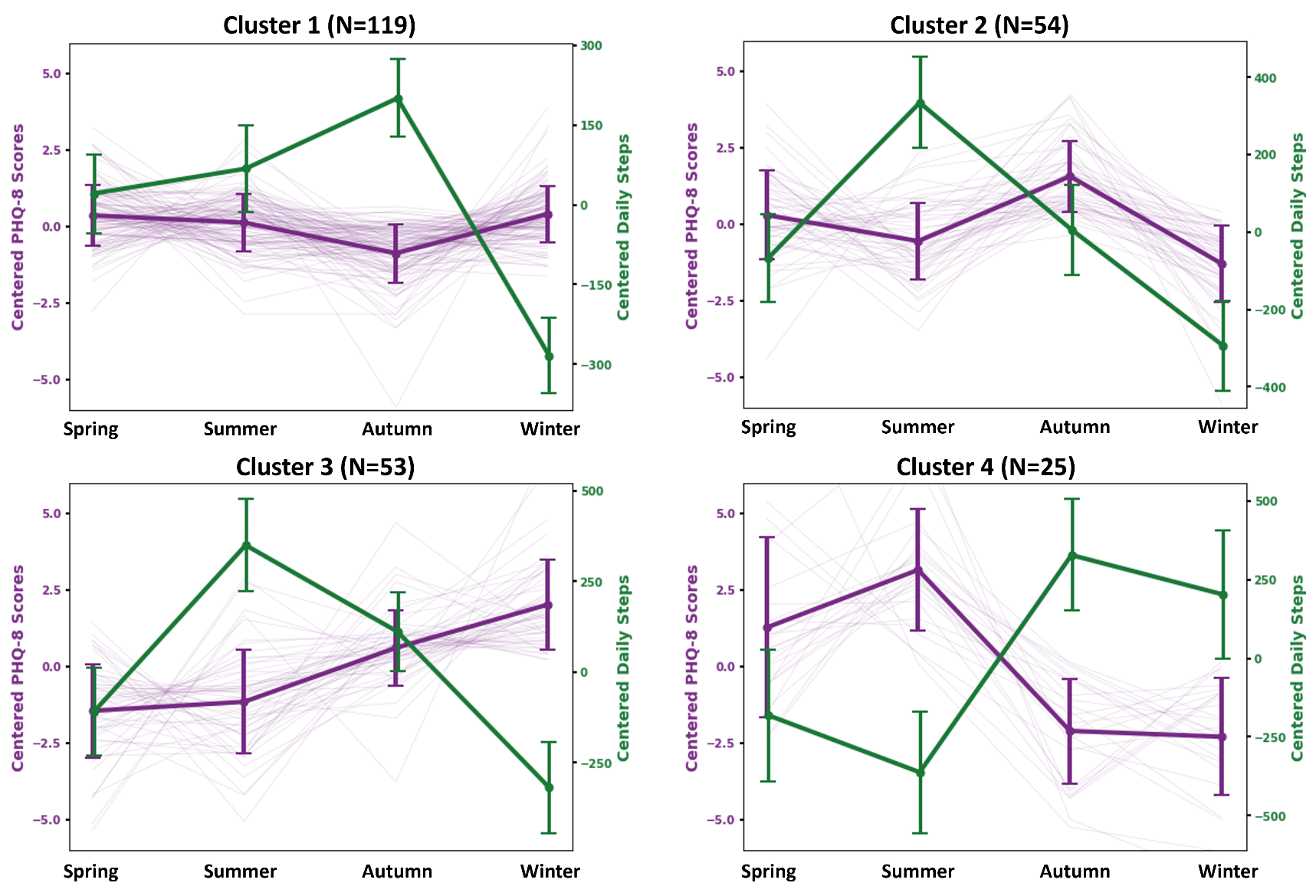


**Supplementary Figure 4. Three distinct patterns of seasonal variations in depression symptom severity (mean-centered PHQ-8 scores, depicted in purple) and corresponding changes in physical activity levels (mean-centered daily steps, depicted in green) in the VUMC study site (Netherlands).**


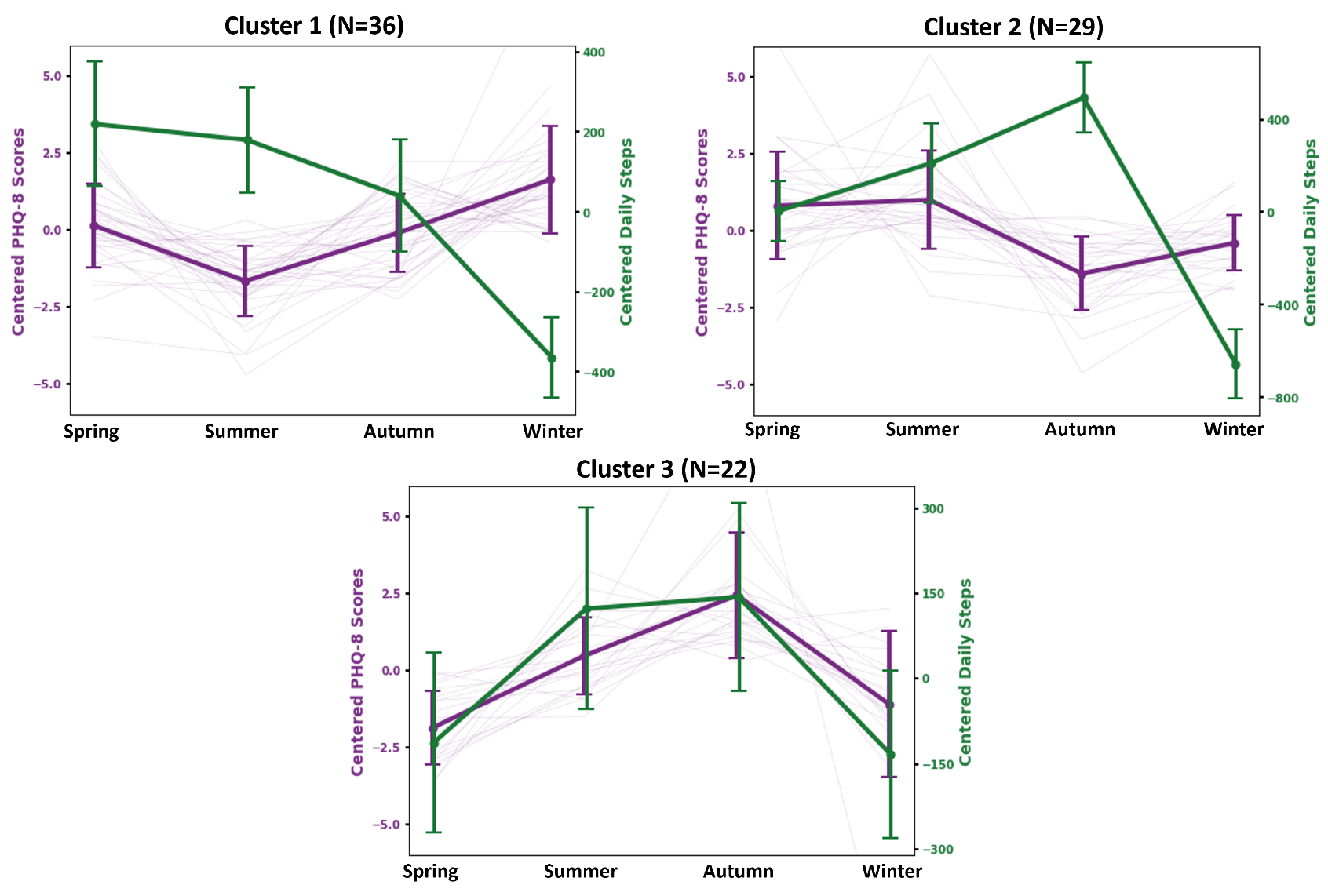

Supplement: Supplementary file 1 — Supplementary [file 44184_2025_125_MOESM1_ESM.docx]
